# Dynamic chromatin accessibility profiling reveals changes in host genome organization in response to baculovirus infection

**DOI:** 10.1371/journal.ppat.1008633

**Published:** 2020-06-08

**Authors:** Xiangshuo Kong, Guisheng Wei, Nan Chen, Shudi Zhao, Yunwang Shen, Jianjia Zhang, Yang Li, Xiaoqun Zeng, Xiaofeng Wu

**Affiliations:** 1 Institute of Sericulture and Apiculture, College of Animal Science, Zhejiang University, Hangzhou, China; 2 Shanghai Jiayin Biotechnology, Shanghai, China; CNRS UMR 7261, Université François-Rabelais, FRANCE

## Abstract

DNA viruses can hijack and manipulate the host chromatin state to facilitate their infection. Multiple lines of evidences reveal that DNA virus infection results in the host chromatin relocation, yet there is little known about the effects of viral infection on the architecture of host chromatin. Here, a combination of epigenomic, transcriptomic and biochemical assays were conducted to investigate the temporal dynamics of chromatin accessibility in response to Bombyx mori nucleopolyhedrovirus (BmNPV) infection. The high-quality ATAC-seq data indicated that progressive chromatin remodeling took place following BmNPV infection. Viral infection resulted in a more open chromatin architecture, along with the marginalization of host genome and nucleosome disassembly. Moreover, our results revealed that chromatin accessibility in uninfected cells was regulated by euchromatic modifications, whereas the viral-induced highly accessible chromatin regions were originally associated with facultative heterochromatic modification. Overall, our findings illustrate for the first time the organization and accessibility of host chromatin in BmNPV-infected cells, which lay the foundation for future studies on epigenomic regulation mediated by DNA viruses.

## Author summary

As a well-studied arthropod-specific double-stranded DNA virus, Bombyx mori nucleopolyhedrovirus (BmNPV) is a representative member of baculoviruses. BmNPV infection results in significant host chromatin marginalization, which has also been found in most DNA viruses. However, the effects of baculovirus infection on the organization and accessibility of host chromatin are poorly understood. Here, by using ATAC-seq, we show that DNA virus BmNPV infection gradually remodels the accessibility of host chromatin. ATAC-seq data reveal that the marginalized host chromatin is a more accessible architecture along with the depletion of multi-nucleosome depositions. Moreover, our findings suggest the increased accessibility regions are regulated by the facultative heterochromatic modification. Overall, we provide a novel understanding of molecular mechanisms by which baculovirus and DNA viruses alter the organization of host chromatin in epigenomic regulation.

## Introduction

DNA viruses are nucleic acid-based obligate intracellular microorganisms, which commonly replicate and assemble in the host nucleus. In the process of infection, the viral genome amplifies in an independent replication compartment and the host genome is marginalized to the nuclear periphery [1–6]. A number of viral proteins can hijack host factors to bind with the gene control elements, which result in large-scale re-organization of the host genome 3D architecture to reprogram gene expression and modulate cell state [7]. Such extensive and conserved changes contribute to modifying and adapting the host intranuclear environment toward a suitable circumstance for efficient viral replication and transcription. However, the effects of DNA virus infection on host genome organization are poorly explored. Therefore, it is fundamental to examine the dynamic changes in host nuclear organization, especially the chromatin accessibility and architecture, for that will facilitate the understanding of the epigenomic manipulation of host chromatin by DNA viruses.

Baculoviruses are a large group of invertebrate-specific DNA viruses with rod-shaped nucleocapsids, which play vital roles in insect pest control and heterologous protein expression [8]. Bombyx mori nucleopolyhedrovirus (BmNPV), a representative member of alphabaculoviruses, is a major pathogen of silkworm *Bombyx mori*, which can cause severe damage to the sericulture industry around the world [9]. BmNPV possesses a ~128kb double-stranded DNA genome, and this genome is assembled within a lipid membrane-enveloped capsid [10]. Similar to other baculoviruses, BmNPV also produces two different phenotypic virions that contain identical genetic material in the life cycle, namely, budded virions (BVs) and occlusion-derived virions (ODVs). The mulberry silkworm is an important economic insect for silk industry, and has abundant and integrated genomic information. Thus, silkworm has been developed as a model organism of lepidoptera insects in the fields of entomology and basic biology. Meanwhile, the interaction between BmNPV and silkworm has also been utilized as a powerful tool to investigate the baculovirus pathology and the baculovirus-mediated lepidoptera pest control.

In the course of baculovirus infection, viral DNA is synthesized in the virogenic stroma (VS), and the host chromatin is marginalized as a result of VS expansion [6, 11]. VS is enriched in proteins essential for viral genome replication and gene transcription, as well as the newly synthesized viral DNA, mRNA and assembled nucleocapsids [8]. In addition to VS formation and host chromatin marginalization, a number of changes have been observed in nuclear organization and architecture in the process of baculovirus infection. For example, P10 protein, a very late hyper-expressed viral protein, causes the formation of nuclear vermiform fibrous structures and is involved in several viral infection processes [12, 13]. Moreover, baculovirus infection induced nuclear actin polymerization disrupted the integrity of nuclear membrane, which is essential to facilitate the nuclear export of nucleocapsids [14]. The maturation of occlusion body within the nucleoplasm involves a complex process, including the formation of intranuclear microvesicles, envelopment of nucleocapsids, and embedding of ODVs into polyhedral [15]. Obviously, baculovirus infection causes a series of intranuclear structural changes, such as nuclear actin accumulation, nuclear membrane disruption, vermiform fibrous structures formation and ODVs maturation.

Although baculovirus infection caused host genome marginalization has been characterized, the states of chromatin accessibility and organization remain largely unknown, even though they have been mentioned in a previous study [16]. Little information is available at present due to the lack of genome-wide methods for analysis. Typically, assay of transposase-accessible chromatin using sequencing (ATAC-seq) is a robust and sensitive method for epigenomic profiling that can also determine the nucleosome position [17, 18]. In ATAC-seq, a hyperactive Tn5 transposase is employed, which allows to simultaneously fragment and integrate the sequencing adapters preferentially in functional regions with accessible chromatin. The ATAC-seq has been widely adopted to investigate the disruption of transcription termination triggered by lytic herpes simplex virus 1 infection, as well as CD8+ T-cell responses to chronic and acute viral infections [19–21]. Therefore, this technology enables us to identify the dynamic changes in chromatin accessibility and architecture during various viral infection processes.

In order to better understand how baculovirus manipulates the host genome, in this report we describe the systematic exploration of the dynamic changes in silkworm chromatin during BmNPV infection by using a combination of epigenetic, transcriptomic, and biochemical assays. According to our results, the host chromatin was remarkably marginalized at the very late stage of infection. Furthermore, ATAC-seq was employed to characterize genome organization, especially for the DNA accessibility of control as well as BmNPV-infected cells at designated time points (8, 24, and 48 hours post infection, h p.i.). As the viral infection progressed, the numbers of differentially accessible peaks increased, and there were more accessible regions in the marginalized host chromatin, with disappeared multi-nucleosome deposition. Finally, we compared the public ChIP-seq data with our ATAC-seq data, which indicated that histone epigenetic modifications were involved in the organization of host genome. Taken together, these results not only illustrate the global profiling of host chromatin accessibility and architecture during BmNPV infection, but also shed new light on the regulation of host epigenomic dynamics in response to baculovirus infection. Meanwhile, our new genome-scale observations directly reveal the organization of marginalized chromatin, which will greatly advance the research of DNA viruses in this aspect.

## Results

### Alterations of host chromatin location induced by BmNPV infection

**Fig 1:**
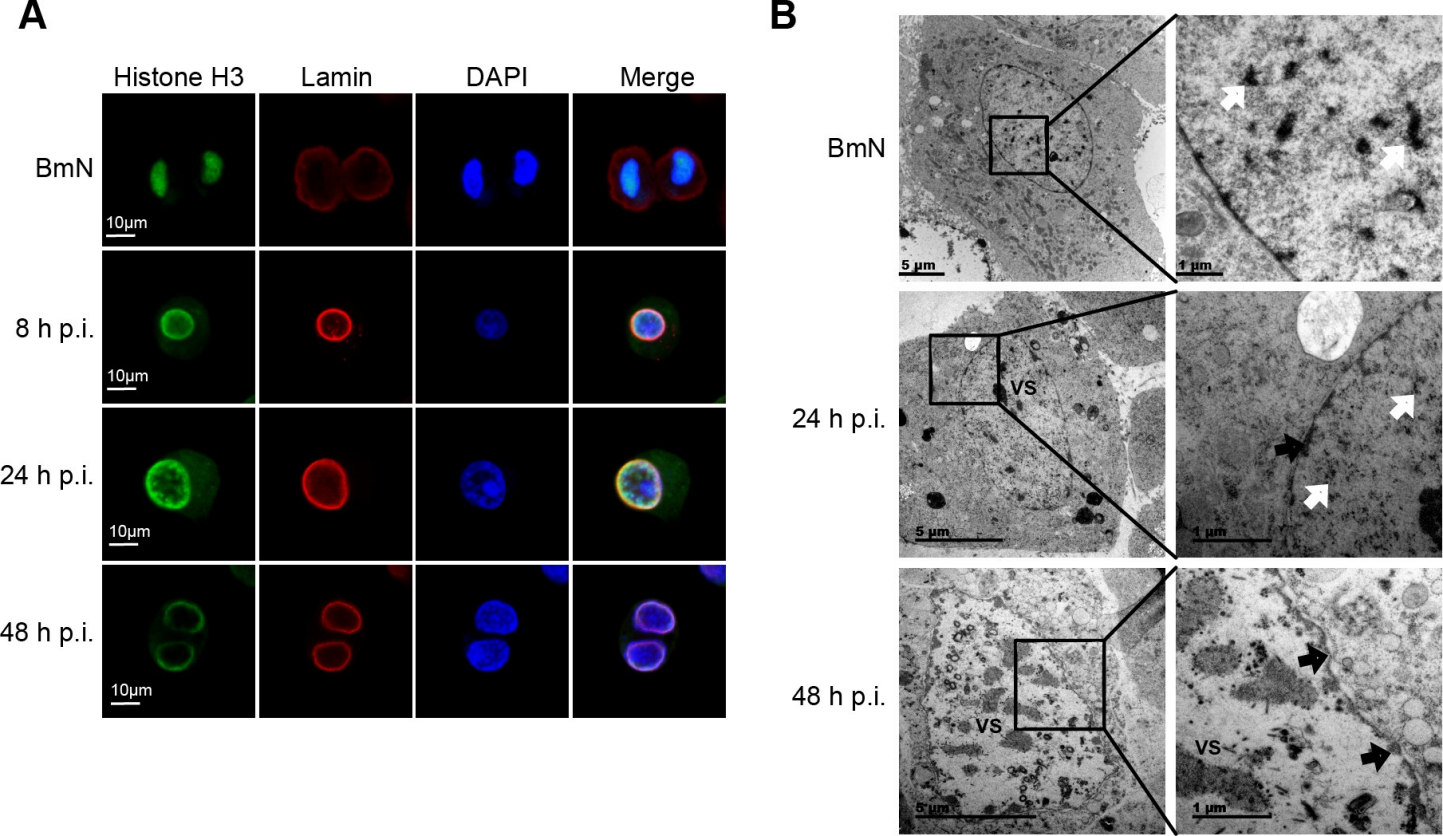
Dynamic locations of the host chromatin. **(A)** Immunofluorescence analysis by confocal microscopy. Fixed BmN cells and BmNPV-infected cells were incubated with anti-histone H3 and anti-lamin Dm0 antibody, followed by treatment with secondary Alexa 488-conjugated antibody (green) and Alexa 546-conjugated antibody (red), respectively. DAPI was used to indicate the location of DNA (blue). The presented cells are representative of a large number of cells. **(B)** HPF/FS-TEM analysis of uninfected BmN cells and BmNPV-infected cells. Over the course of viral infection, electron density (white arrows) in the nucleoplasm was gradually reduced concomitantly with the expansion of virogenic stroma (VS). Black arrows indicate the marginalized chromatin.

It is well known that the baculovirus-induced VS is the region responsible for both viral DNA replication and nucleocapsid assembly [11]. Although the marginalization of host chromatin concomitant with VS expansion has been observed, with the heterologously expressed recombinant histone H4 being used as a chromosome marker [6], the location of endogenous histone remains unknown. Histone H3 is more evolutionarily conserved than histone H4 in eukaryotes; therefore, we used a commercial histone H3 antibody to analyze host chromatin distribution using immunofluorescence microscopy. During metaphase of BmN cells, histone H3 was colocalized with the concentrated DNA (**Fig 1A**), suggesting that histone H3 was integrated with host chromatin and can be used as an effective marker of chromosome distribution. Following VS expansion from 8 to 48 h p.i., the host genome was gradually located at the lamin-labeled inside region of nuclear membrane (**Fig 1A**). To further characterize the alteration of nuclear ultrastructure, we used the high-pressure freeze (HPF) and freeze substitution (FS) techniques in combination with transmission electron microscopy (TEM) [22] to observe the infected intranuclear organization. As shown in **Fig 1B**, VS occupied the nuclear center, and numerous electron-dense depositions were distributed in the nucleoplasm around VS at 24 h p.i. By 48 h p.i., VS had gradually expanded concurrent with the marginalization of electron-dense depositions around the perinuclear membrane (**Fig 1B**), indicating that these depositions were host chromatin. The central region of the nucleus was left for nucleocapsid assembly and ODV envelopment. Taken together, these results demonstrated that BmNPV infection resulted in host chromatin extruded into the inside region of nuclear membrane, which was thought to change the host genome organization.

### The landscape of chromatin accessibility in BmN cells during BmNPV infection

In eukaryote cells, genome is dynamic and non-randomly organized in the nucleus. Therefore, it was surmised in this study that DNA accessibility was dynamically remodeled during host chromatin marginalization. To confirm our hypothesis, we applied ATAC-seq technology to investigate the effects of BmNPV infection on the global host chromatin accessibility in BmN cells. According to our ATAC-seq data, consistent with data from the *Drosophila melanogaster* embryonic ATAC-seq profiles [23], BmN cells exhibited an expected nucleosomal insert size pattern (**S1A Fig**), which was different from the results in mammals [24]. Besides, the chromatin accessibility profiles were highly correlated between two replicate samples (R = 0.94–0.96), indicating the reproducibility and reliability of our data in assessing chromatin accessibility (**S1B Fig**). The accessible regions were visualized and quantified, which suggested that the obtained data were of high-quality with a strong and stable signal density to background (**S1C Fig.**).

**Fig 2:**
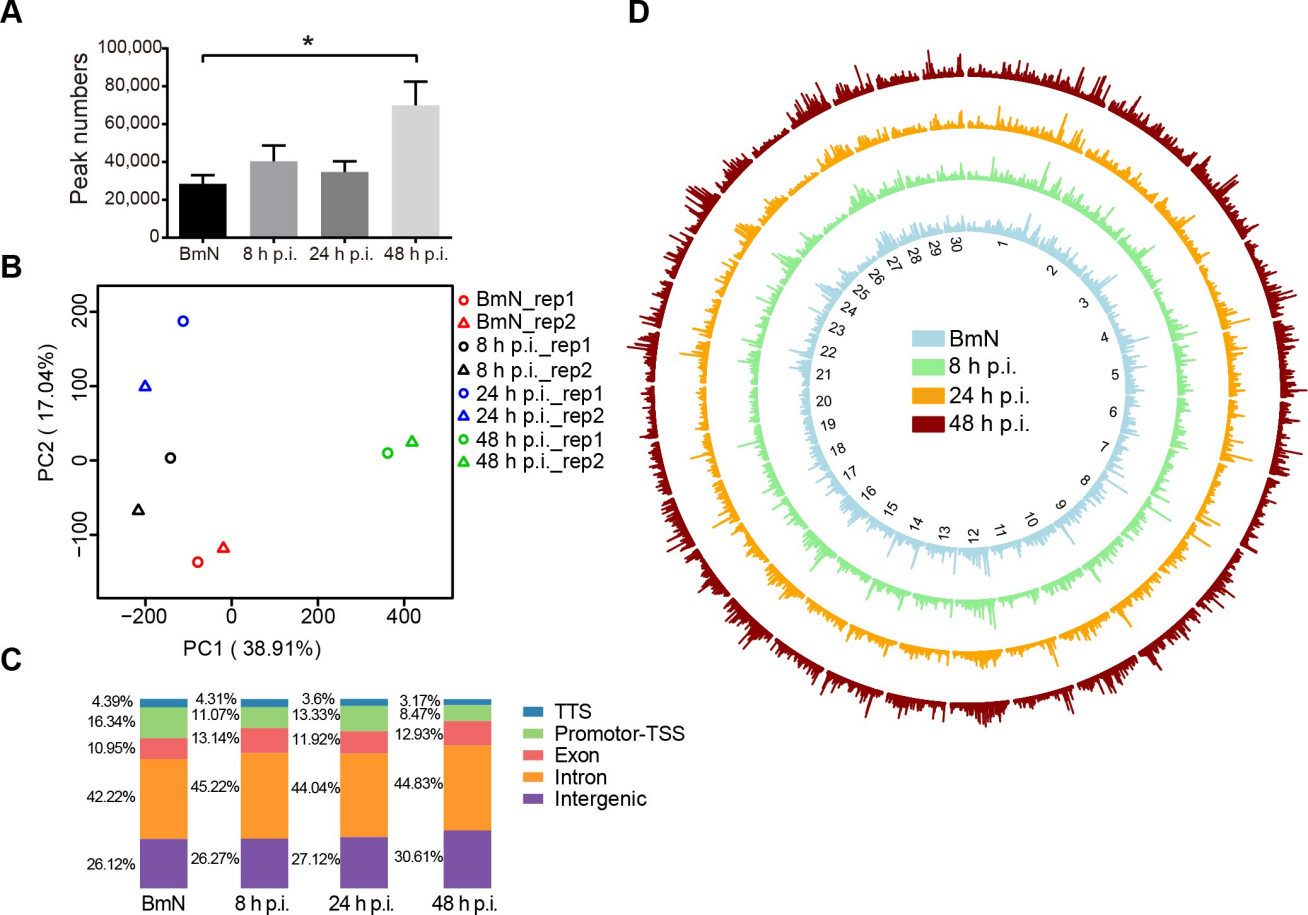
Landscape of DNA accessibility in BmN cells and BmNPV-infected cells. **(A)** The accessible peak numbers of each sample. Student’s *t*-test was performed between BmN samples and 48 h p.i. samples. **(B)** PCA of peak accessibility in all samples. Each symbol represents an independent biological replicate. PC1 (38.91%) and PC2 (17.04%). **(C)** Distribution of genomic features of all accessible regions. Five annotations were examined, transcription terminal site (TTS), promoter-transcription start site (TSS), exon, intron, and intergenic. **(D)** Circle diagram depicting the chromatin accessibility of the 30 longest scaffolds.

Statistical analysis revealed that the number of accessible peaks was higher in BmNPV-infected cells than in control group, which was especially significant at 48 h p.i. (**Fig 2A**). In addition, principal components analysis (PCA) also exhibited a significantly positive similarity among the open chromatin landscapes in each group and progressive changes in BmNPV-infected cells, which suggested that viral infection resulted in substantial chromatin remodeling (**Fig 2B**). To further characterize the genomic functional elements of accessible regions, HOMER [25] was conducted to assess the genomic features, and it was found that about 70% ATAC-seq peaks were predominately enriched in the intergenic and intron regions (**Fig 2C**) during infection. In contrast, the enrichment of promoter-TSS gradually decreased from 18 to 8% as the viral infection progressed (**Fig 2C**). Additionally, the genome-wide chromatin accessibility was visualized through comparing those regions of the top 30 size scaffolds among all samples (**Fig 2D**). This circle diagram clearly displayed the radical and global alterations of chromatin accessibility in these samples in response to BmNPV infection (**Fig 2D**). To sum up, these ATAC-seq data demonstrated that BmNPV infection induced dynamic changes in the chromatin accessibility.

### Analysis of differentially accessible peak reveals progressive host genome remodeling

**Fig 3:**
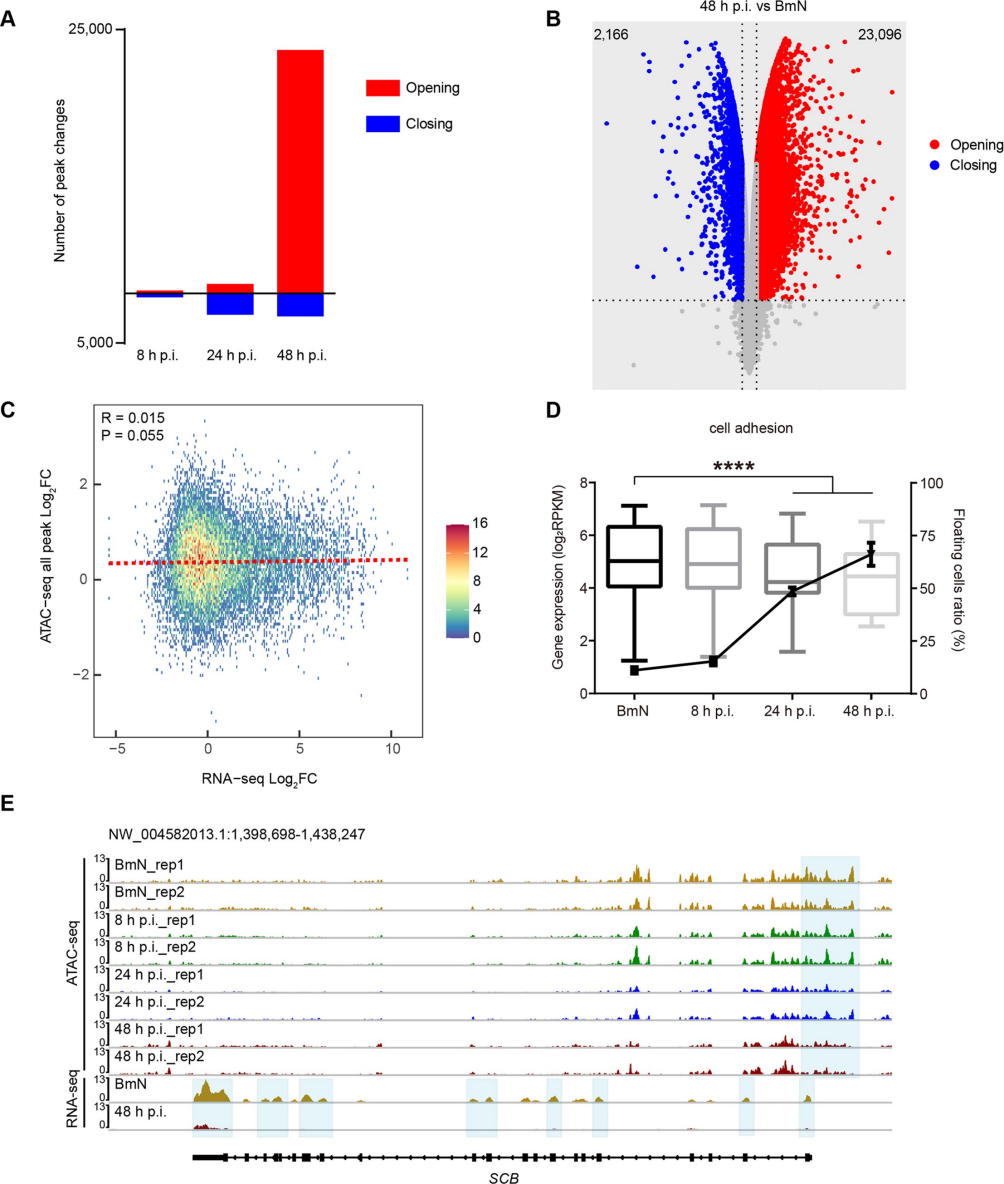
Differentially accessible peaks analysis. **(A)** The number of differentially accessible peaks at 8, 24, and 48 h.p.i. **(B)** Volcano plot of differentially accessible peaks between BmN cells and the 48 h p.i. group. **(C)** Correlation of chromatin accessibility at all peak and gene expression between BmN cells and the 48 h p.i. group. **(D)** Boxplot of the expression levels of cell adhesion associated genes and the proportion of floating cells during viral infection. Two-way ANOVA was performed to analyze the significance of gene expression levels between BmN cells, and the 24 and 48 h p.i. groups. Student’s *t*-test was performed for floating cell data between BmN cells, and 24 and 48 h p.i. samples. **(E)** Normalized ATAC-seq and RNA-seq profiles at *SCB* loci. Shaded regions are representative of a decline by 48 h p.i.

To identify the specific dynamic changes in the chromatin accessibility landscape in response to viral infection, limma [26] was used to analyze the differentially accessible peaks (DAPs). We found that massive chromatin remodeling occurred at 24 and 48 h p.i., and less remodeling at 8 h p.i. (**Fig 3A**). Notably, over 23,000 ATAC-seq peaks gained accessibility at 48 h p.i., and they were specifically enriched in intergenic and intron regions, but less enriched in promoter-TSS and exon elements. In contrast, peaks that were more accessible in 8 and 24 h p.i. groups were preferentially enriched in promoter-TSS and exon elements (**S2A Fig**), suggesting that these elements might have greater effects on regulating gene expression at early and late infection phases. Among peaks that were accessible in any of these groups, few (n = 642) were differentially accessible between the control and 8 h p.i. group (**S2B Fig**); however, such figure was dramatically changed to about 3,000 DAPs between the control and 24 h p.i. group (**S2B Fig**), and over 25,000 DAPs were identified between the control and 48 h p.i. group (**Fig 3B**). As shown in **Fig 3C**, we found that accessibility of all peak has no significant correlation with expression of genes. In addition, the relationships between gene expression and chromatin accessibility were weak across genes that gained or lost accessibility in promoter-TSS elements at 8 and 24 h p.i. (**S2C and S2D Fig**), indicating that the accessibility at promoter-TSS was not a major element responsible for transcriptional regulation in response to viral infection at the early or late stages. However, when the infection progressed to 48 h p.i., gene expression was highly regulated by chromatin accessibility at promoter-TSS elements (**S2E Fig**).

**Fig 4:**
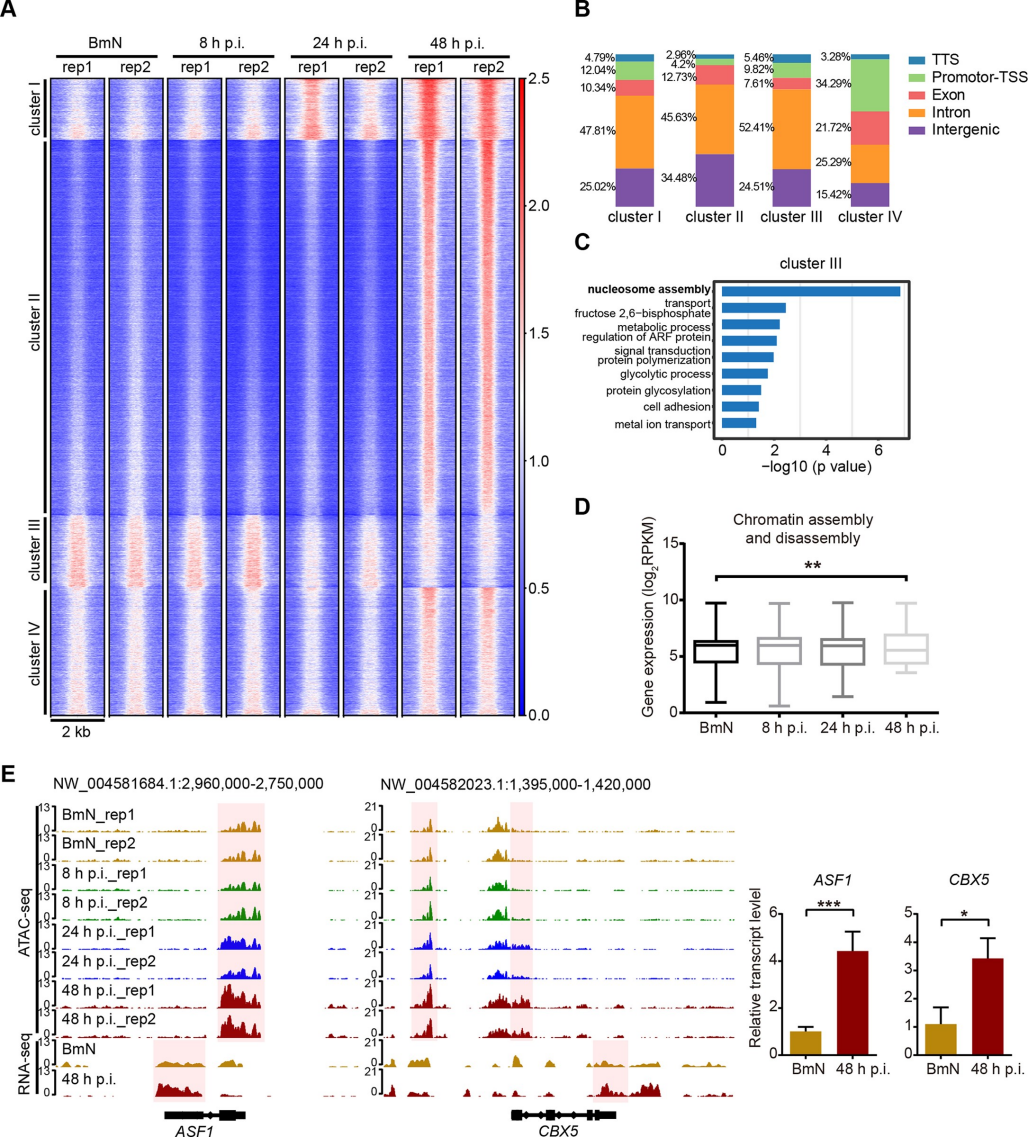
Genome-wide increase in DNA accessibility is associated with chromatin assembly and disassembly of different GO group clusters. **(A)** Heatmap of 61,421 elements. Each row represents one element. Peaks are grouped using K-means clustering. The numbers on the right indicate relative signal intensity. **(B)** Distribution of genomic features of four clusters. **(C)** Biological processes (GO terms) enriched in genes which corresponding to the cluster III region. **(D)** Boxplot of the expression of genes related to chromatin assembly and disassembly. Two-way ANOVA was performed between BmN and the 48 h p.i. groups. **(E)** Normalized ATAC-seq and RNA-seq profiles at *ASF1* and *CBX5* loci. Shaded regions representative an increase at 48 h p.i.

To further identify the roles of these DAPs in viral infection, the functions of DAPs-corresponding genes were annotated based on gene ontology (GO) analysis (**S3A–S3F Fig**). Typically, GO term related to innate immune response was enriched in the opening sites at 8 h p.i. (**S3A Fig**), which showed that the innate immune pathways were activated at the early stage to defend against viral infection. For instance, the promoter-TSS and upstream distal elements in the *LOC101735468*, a *toll-like receptor 5* homologue in *Bombyx mori*, gained accessibility at 8 h p.i. (**S2F Fig**). These findings were consistent with previous studies reporting that baculovirus infection activated the host Toll innate immune pathway in both mammals and invertebrates [27–29]. Moreover, GO term for cell adhesion was concurrently enriched at sites that lost accessibility at 24 and 48 h p.i, which was also found in the cluster III regions (**Fig 4C** and **S3D and S3F Fig**). By comparing the RNA-seq data, and the gene loci that lost density, we observed a statistically significant decline in gene expression during viral infection (**Fig 3D**). In addition, the cell detachment assay [30] also revealed a progressive increase in the proportion of floating cells during BmNPV infection (**Fig 3D**). Further, the loci of *SCB (LOC101746113)*, an *integrin alpha PS3* (*Drosophila melanogaster*) homologue, exhibited a deceasing trend in both DNA accessibility and gene expression (**Fig 3E**). The above results collectively revealed that BmNPV infection epigenetically suppressed the expression of cell adhesion genes and induced host cell detachment.

Taken together, these findings supported the notion that baculovirus infection dramatically remodeled the host chromatin accessibility. In addition, our data served as the effective sources to investigate virus-host interaction by combining epigenome with transcriptome.

### Chromatin accessibility broadly increased at the very late stage of BmNPV infection

In contrast to a previous study reported that no alteration in the host chromatin structure [16], our findings suggested that viral infection induced extensive changes in host chromatin architecture. To further dissect more details about chromatin organization remodeling, 61,421 specific regions across the genome from all samples merged with high ATAC-seq signals were examined. The corresponding K-means clustering analysis revealed four distinct clusters of regulatory elements, suggesting that BmNPV infection gave rise to distinctly different epigenetic signatures from those of control (**Fig 4A**). After assessing the genomic features of these cluster peaks, only 4.2% peaks were found to be distributed in promoter-TSS elements of cluster II, which was substantially less than other clusters (**Fig 4B**).

Cluster I was comprised of 5,936 peaks that gradually gained accessibility as a response to viral infection; and cluster IV contained 12,333 peaks with increasing accessibility by 48 h p.i. These regions might reflect the host chromatin response activated by BmNPV infection. GO analysis showed that the peaks in cluster I were highly enriched in translation and transcription related genes, such as those involved in translation, post-translational protein modification, and transcription (DNA-templated); in addition, regulation of transcription (DNA-templated) was enriched in cluster IV (**S4A and S4C Fig**). These enriched GO terms were also observed at sites that gained accessibility at 24 and 48 h p.i. groups (**S3C and S3E Fig**), which indicated that BmNPV infection activated the host transcription and translation systems to facilitate viral gene expression. For example, the regulatory elements associated with the promoter-TSS region of *ribosomal protein L21* (*RpL21*), a component of the large ribosomal subunit, were gradually up-regulated in cluster I (**S4D Fig**). Another example from cluster IV includes the upstream distal element of gene *E2F transcription factor 1*, *E2F1*, was up-regulated at 48 h p.i. (**S4E Fig**). The protein encoded by *E2F1* is a transcription factor involved in several host responses induced by baculovirus infection, such as, regulation of apoptosis [31] related gene expression.

Cluster II was comprised of more than 36,000 peaks that were specifically accessible in 48 h p.i. group, but had a very low signal in both the control, 8 and 24 h p.i. groups (**Fig 4A**). This observation suggested that viral infection led to numerous regions that uniquely gained accessibility at 48 h p.i., and such result was similar to the differential analysis showing that more than 23,000 peaks gained accessibility at 48 h p.i. (**Fig 3A**). Although fewer promoter-TSS elements were enriched in this cluster, the genes close to these elements were highly expressed in 48 h p.i. group as evidenced by comparing the RNA-seq data of two representative GO terms (**S4F and S4G Fig**). Consequently, the above results indicated that peaks in cluster II broadly and uniquely gained accessibility at 48 h p.i.

Cluster III consisted of 6,985 regions that gradually lost accessibility during viral infection (**Fig 4A**). GO analysis showed that elements in this cluster were highly enriched for genes associated with nucleosome assembly, which was also observed in GO functions that lost accessibility at 24 and 48 h p.i. (**Fig 4C** and **S3D and S3F Fig**). Nonetheless, the expression of nucleosome assembly-associated genes was not significantly down-regulated, as evidence by the RNA-seq data (**S4H Fig**). Surprisingly, the GO term associated with chromatin assembly or disassembly was enriched in regions that gained accessibility at 48 h p.i. (**S3E Fig**). Transcriptomic analysis revealed that the expression of these genes was remarkably up-regulated in 48 h p.i. group (**Fig 4D**). Prime examples of this group including elements in *CBX5 (LOC101740470)* and *ASF1 (LOC101742242)* loci, encoding chromobox protein homolog 5 (CBX5) and histone chaperone asf1(ASF1) respectively, became strongly accessible at 48 h p.i. (**Fig 4E**). qRT-PCR results confirmed that these two genes were significantly up-regulated at 48 h p.i. (**Fig 4E**). CBX5 is a heterochromatin component that drives heterochromatin distribution to the inner nuclear membrane through interacting with lamin-B receptor (LBR) [32, 33]. Moreover, ASF1 is a histone chaperone that facilitates histone deposition, exchange and removal in the process of nucleosome disassembly [34, 35].

Taken together, these results indicated that BmNPV infection induced certain genome regions to gain accessibility and these regions positively regulated gene expression at 48 h p.i.

### Genome-wide increase in DNA accessibility is accompanied by nucleosome landscape depletion

**Fig 5:**
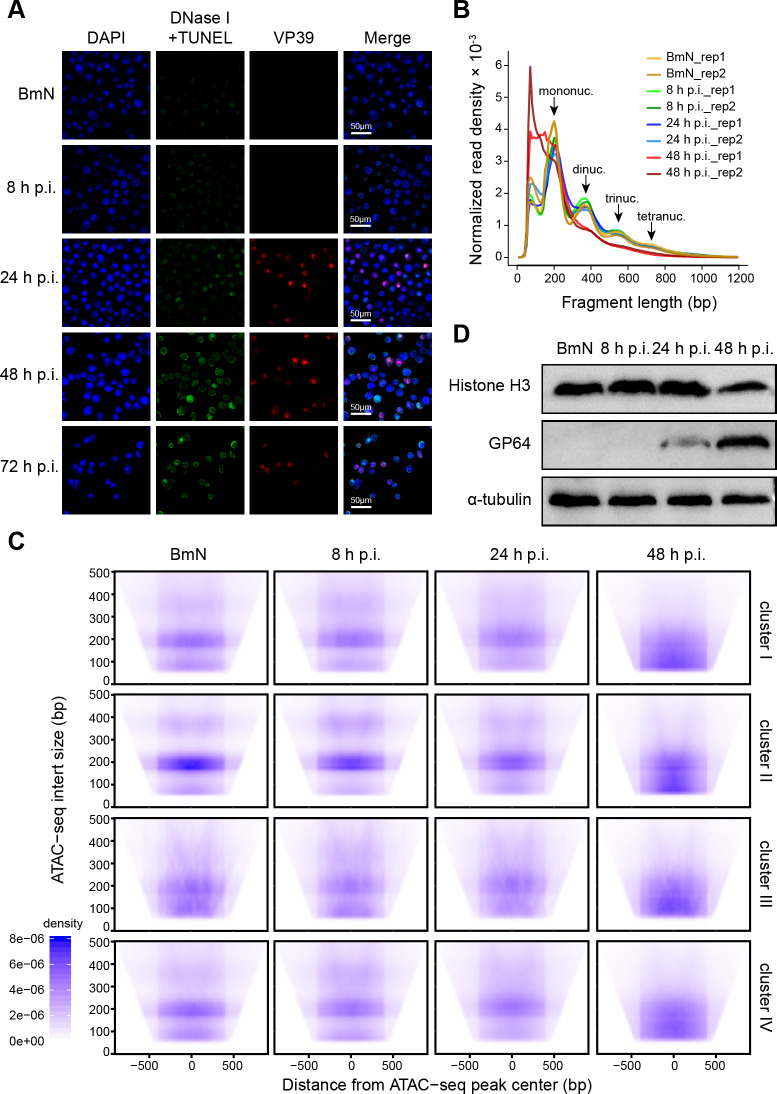
Widespread increase in DNA accessibility is accompanied by nucleosome disassembly. **(A)** DNase I TUNEL assay. Immunostaining of VP39 indicates VS regions. **(B)** Fragment length distribution plots of ATAC-seq samples. **(C)** ATAC-seq insert size for four clusters visualized by V-plots. The density of insert size is shown by aggregation based on distance from the ATAC-seq peak center. **(D)** BmN cells and BmNPV-infected cells were analyzed by western blot with the indicated antibodies.

To further investigate the genome-wide chromatin organization remodeling, we carried out the *in vivo* DNase I TUNEL assay [36] to detect the genomic chromatin accessibility to DNase I. As revealed by our results, only weak TUNEL signal was detected in BmN cells, and the fluorescence intensity gradually increased as the viral infection progressed (**Fig 5A**). These results verified our conclusion that chromatin accessibility broadly increased following BmNPV challenge.

Chromatin organization is a fundamental biological process mediated by nucleosome assembly and disassembly in eukaryotic genomes [37, 38]. Therefore, it was proposed in this study that the viral infection-induced broad increase in DNA accessibility also changed the nucleosome organization. To test this hypothesis, we examined the nucleosome landscape dynamics through analyzing the distribution of fragment lengths. According to our results, expected mono-nucleosomal and multi-nucleosomal insert size patterns were observed both at the early and late stages of viral infection, which was similar to the pattern observed in control group, whereas the multi-nucleosomal insert size distribution obviously decreased at the very late stage of infection (**Fig 5B**). To identify the detailed changes in nucleosome organization, we used the insert size density plot (V-plot) to calculate the visualization of ATAC-seq data. The position of flanking nucleosomes in the control showed an enrichment of short fragments (less than 140 bp) in clusters I, III and IV, together with an enrichment of mono-nucleosomal size fragments (150–200 bp) in cluster II. These results indicated that the chromatin in cluster II was compactly organized by nucleosomes and less accessible, however, the regions in other clusters were accessible in the control (**Fig 5C**). For virus-infected cells at 48 h p.i., the insert size mostly concentrated in the short fragments across all clusters (**Fig 5C**). In cluster II, the short fragments were only enriched at 48 h p.i. (**Fig 5C**), demonstrating that the regions in this cluster lost nucleosome deposition and gained accessibility. Moreover, the expression of histone H3 was down-regulated at 48 h p.i. (**Fig 5D**), which was also observed in BmNPV-infected *Bombyx mori* pupae [39]. The above findings strongly supported our hypothesis that viral infection induced widespread increase in chromatin accessibility, along with the large-scale nucleosome disassembly.

### Epigenetic modifications are involved in chromatin accessibility profiles maintenance

**Fig 6:**
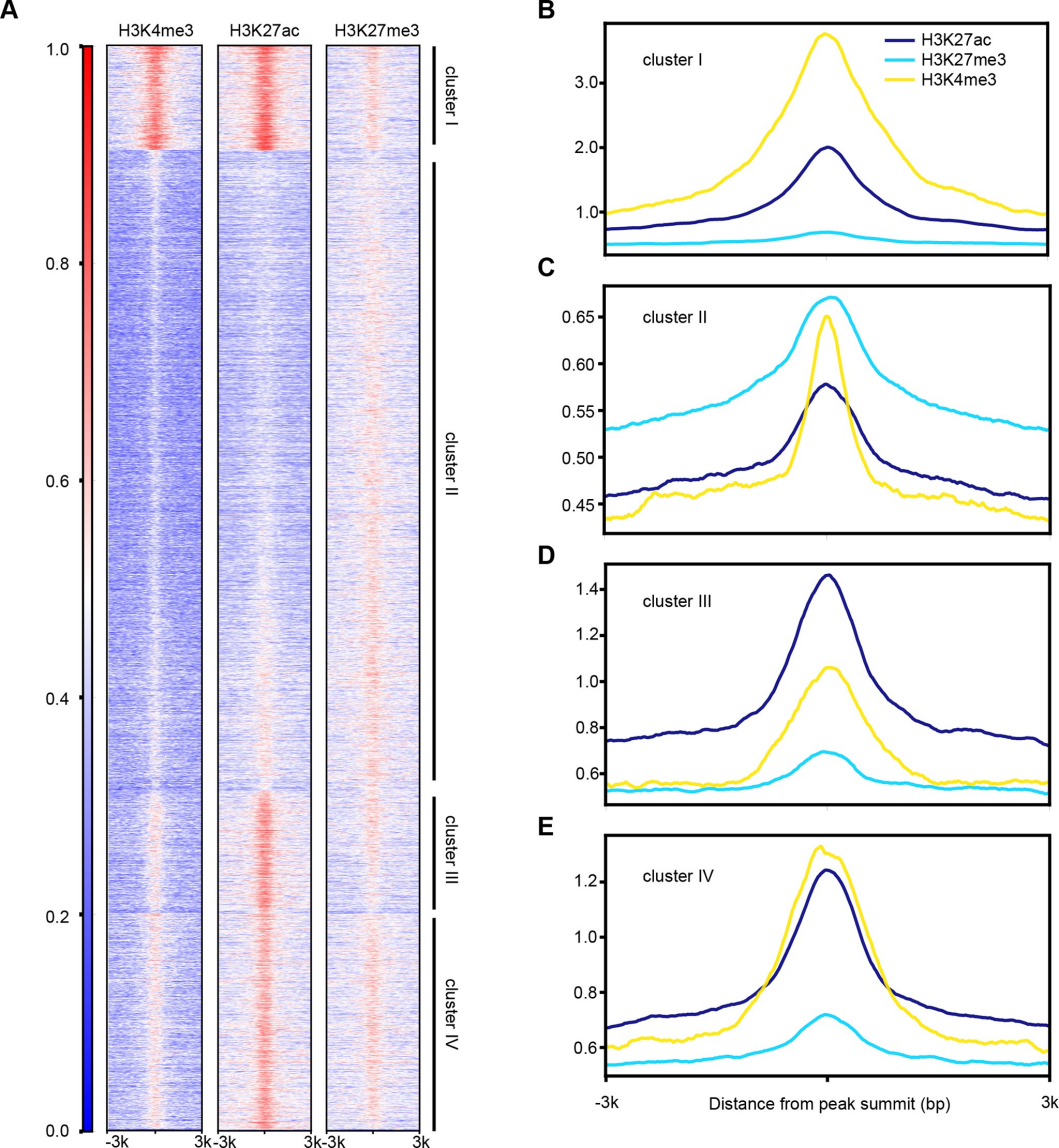
Histone modifications regulate the maintenance of DNA accessibility. **(a)** Heatmap of H3K4me3, H3K27ac and H3K27me3 ChIP-seq data. The numbers on the left indicate relative signal intensity. **(b-e)** Average of normalized ChIP-seq read counts of four clusters.

Previous studies showed that epigenetic modifications of histone H3 in the silkworm were similar to other eukaryotes, and H3K4me3, H3K27ac were most prominent euchromatin markers, whereas H3K9me3 and H3K27me3 represented heterochromatin regions in BmN cell line [40–42]. In eukaryotes, epigenetic modifications strictly regulate chromatin organization, nucleosome assembly and transcription. On this account, ChIP-seq data [41, 42] obtained from public databases were utilized to test the relationship between histone H3 epigenetic modifications and chromatin accessibility. Comparing the epigenetic modification profiles of three methylated or acetylated forms of histone H3 with the chromatin accessible pattern of controls, which suggested significant overlaps between H3K4me3 (66.15%) and H3K27ac (48.37%) with ATAC-seq peaks; however, only 17.93% regions of H3K27me3 overlapped with the accessible regions (**S5A–S5C Fig**). Moreover, the H3K4me3 and H3K27ac profiles displayed a similar landscape pattern to that of the open chromatin regions, particularly enriched in clusters I, III and IV and lost signal in cluster II (**Fig 6A** and **Fig 4A**). In contrast, H3K27me3 had signals in cluster II regions (**Fig 6A**). Thus, the concentrations of DNA-associated H3K27ac and H3K4me3 were dramatically higher than those of H3K27me3 in clusters I, III and IV sites, whereas that of H3K27me3 was higher in cluster II regions than those of the other two modifications (**Fig 6B–6E**). These results suggested that the open chromatin regions significantly overlapped with H3K4me3 and H3K27ac, indicating that these two euchromatin markers were involved in the accessible chromatin organization. As a heterochromatin marker, however, H3K27me3 participated in the maintenance of epigenetic repression state in cluster II regions in the control, and this cluster was activated at the very late stage of BmNPV infection.

### H3K27ac participates in the genome-wide increase of chromatin accessibility

**Fig 7:**
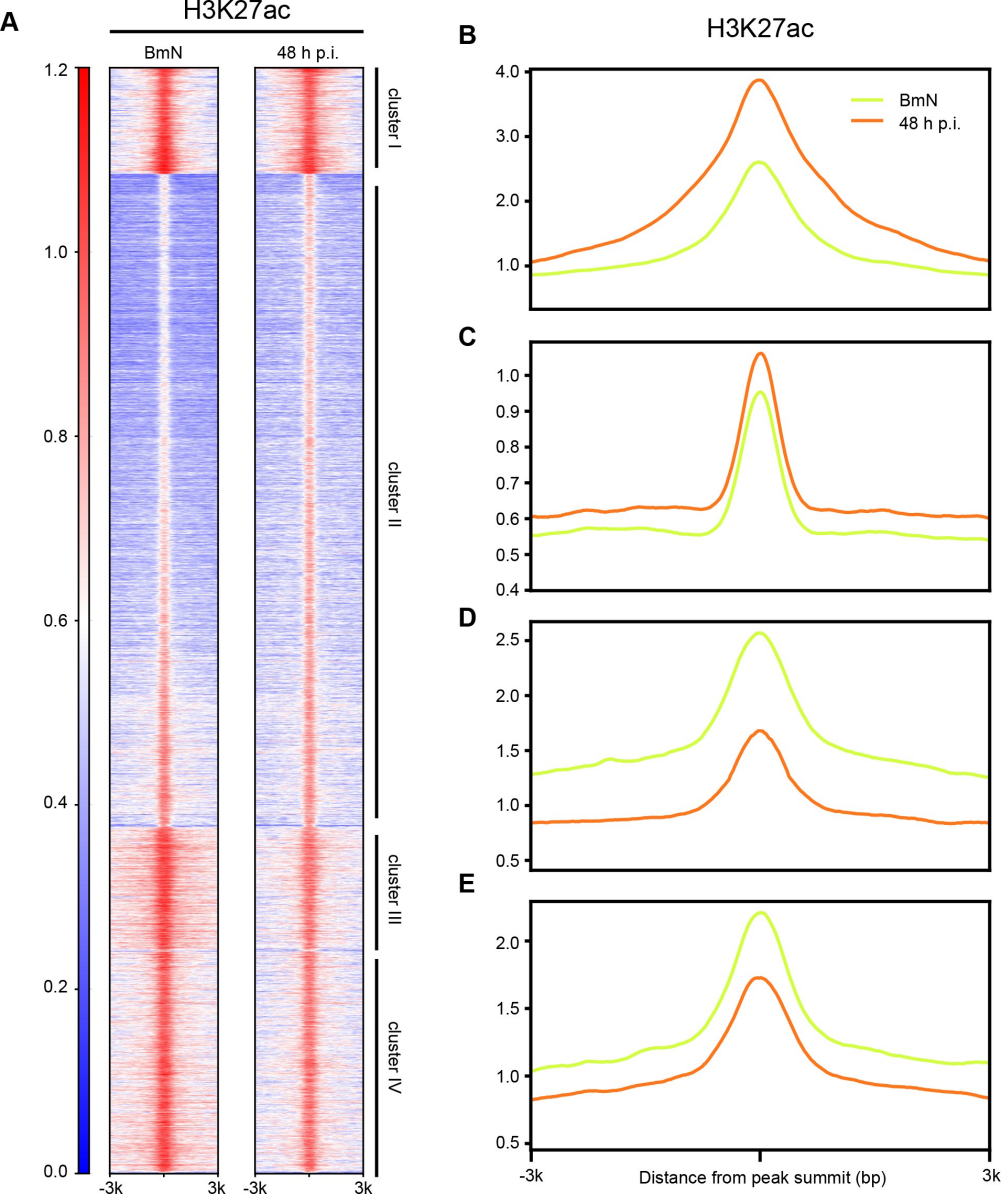
The relationship between H3K27ac and accessibility. **(a)** Heatmap of H3K27ac ChIP-seq data. The numbers on the left indicate relative signal intensity. **(b-e)** Average of normalized ChIP-seq read counts of four clusters.

Subsequently, to determine the functions of histone modifications in regulating chromatin accessibility upon BmNPV infection, we detected the levels of these modifications. As shown in **S5D Fig**, H3K27ac was up-regulated and the other methylated histone forms were down-regulated at 48 h p.i., indicating that H3K27ac might be involved in the BmNPV infection-induced widespread gain accessibility. Thus, ChIP-seq was conducted to test the relationship between H3K27ac and DNA accessibility during virus infection. As expected, H3K27ac in clusters I and II regions had higher signals in 48 h p.i. group than in control group (**Fig 7A–7C**). In contrast, the enrichments of H3K27ac in clusters III and IV sites were markedly higher in BmNPV infected cells than in uninfected cells (**Fig 7A, 7D and 7E**). These results suggested that H3K27ac exerted essential roles in viral-induced the regions of clusters I and II that gained accessibility at the very late stage of infection.

## Discussion

**Fig 8:**
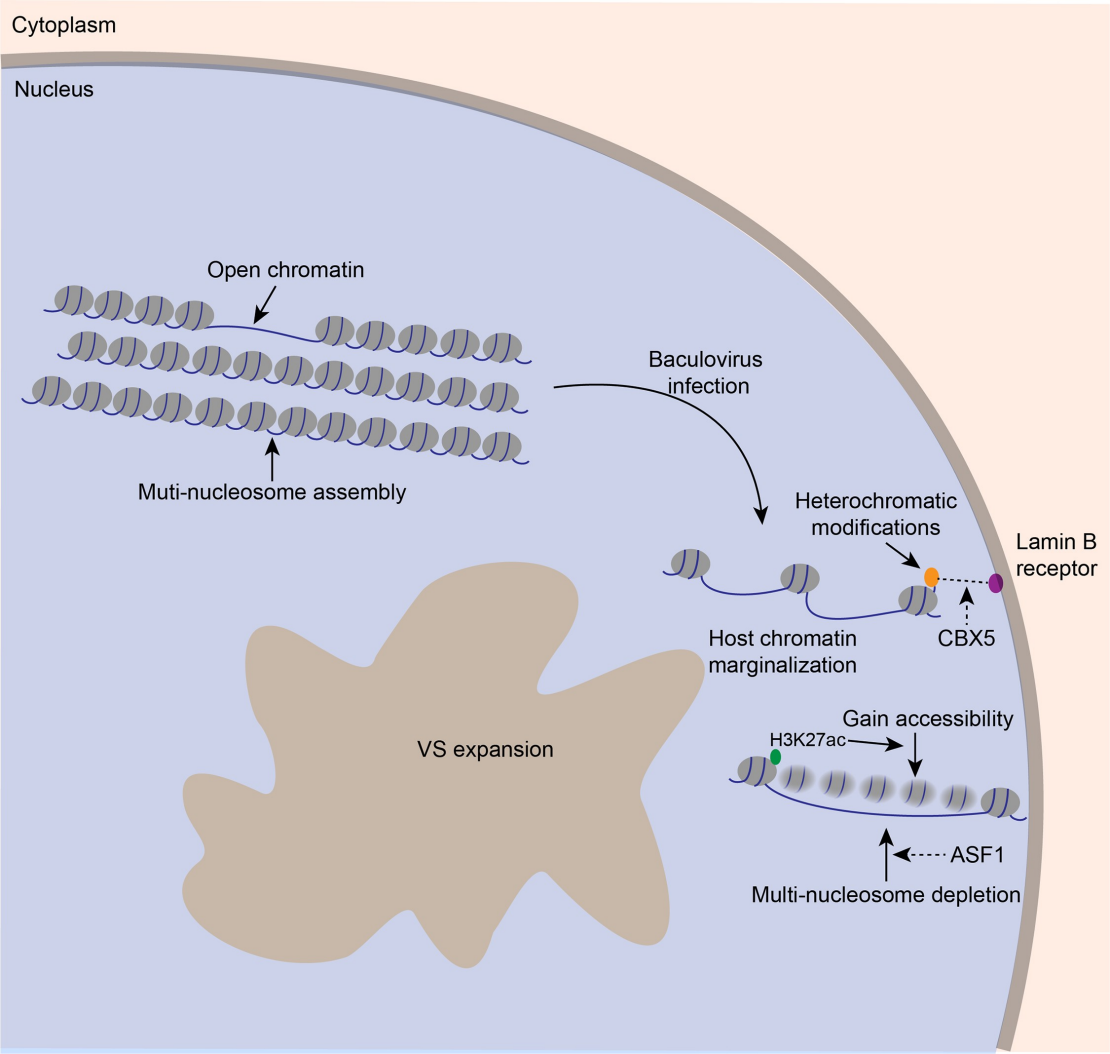
Summary of changes in chromatin organization and accessibility during BmNPV infection. In uninfected BmN cells, the host genome tightly compacted by multi-nucleosome depositions and is accessible in limited locations. During baculovirus infection, host chromatin is marginalized combined with the VS expansion. Up-regulated accessibility and expression at *CBX5* locus indicated that CBX5 is a potential regulator for this process via interacting with the lamin B receptor. At the very late stage of infection, genome-wide regions gain accessibility and ASF1 may mediate the disassembly of multi-nucleosome depositions. This schematic modeled on our data, and dotted line and arrows indicated the possible underlying mechanisms based on extrapolation of data.

In this study, we performed ATAC-seq approach to investigate the BmNPV infection mediated changes in genome organization and accessibility. Epigenome, transcriptome and biochemical assays were carried out to comprehensively examine the host chromatin accessible landscape in response to baculovirus infection. As revealed by our data, radical genome remodeling and a broad increase in chromatin accessibility occurred concomitantly with nucleosome disassembly when the host genome was marginalized during viral infection (**Fig 8**). These results contribute to explaining the effects of viral infection on the dynamic changes in chromatin accessibility and organization, and lay the important basis to explore the virus-host interactions, especially the epigenome mechanism during the viral life cycle.

Many DNA viruses replicate in the intranuclear viral replication compartment (VRC), and viral DNA can directly/indirectly interact with the host genome [43–45]. The expansion of VRC is accompanied by host chromosome marginalization to nuclear periphery and an increase in nuclear volume [1–5]. The baculovirus-caused chromatin marginalization has also been well investigated, however, the molecular mechanism remains largely unknown. Our findings suggested that BmNPV infection increased the accessibility and expression of *CBX5* locus. CBX5, a homologue of *Drosophila* heterochromatin protein 1 alpha, exerts an essential role in the formation and maintenance of heterochromatin architecture [46]. CBX5 contains a chromobox domain, which can directly recognize and bind with heterochromatic modifications [46], as a result, it tethers heterochromatin to the inner nuclear membrane by interacting with the lamin B receptor (LBR) [32, 33]. Therefore, it was proposed in this study that the increased *CBX5* accessibility and expression is involved in the baculovirus-induced chromatin marginalization by employing CBX5-LBR interaction (**Fig 8**). In addition, LBR is a part of the nuclear lamina (NL) and NL closely contacts with specific genomic regions, which is termed as lamina-associated domains (LADs) [47, 48]. NL disruption results in the increased histone H3 pan-acetylation and transcriptional upregulation in LADs [49]. In baculovirus infected cells, viral infection induces the disruption of NL and phosphorylation of lamin B [50]. Thus, it is important for us to understand the relationships between NL, LADs, heterochromatic modifications and chromatin marginalization upon baculovirus infection.

The increased peak numbers and DAPs illustrated that substantial chromatin remodeling occurred in the BmNPV-infected cells. A recent study in yeast reveals that positioning heterochromatin at the nuclear periphery facilitates epigenetic suppression [51]. However, our results suggested that the marginalized host genome was not a condensed organization, instead, it was a more accessible architecture. In addition, nucleosome disassembly was observed accompanying with the widespread increase in chromatin accessibility. This mechanical difference in epigenomic regulation between normal eukaryotic cells and BmNPV-infected cells may be ascribed to the fact that, viral infection fundamentally changes the general location and organization of host genome and the rules of epigenomic organization are altered by BmNPV. The *ASF1* locus encodes a histone chaperone that facilitates histone deposition. Our results confirmed the speculation that the increased accessibility and up-regulated expression at *ASF1* locus participated in the chromatin disassembly at the late infection (**Fig 8**). The alterations of DNA accessibility and nucleosome assembly are suggested to affect the 3D genome structure [52], including chromatin loops, topological associating domains (TADs) and the links between their spatial positioning [53–55]. The chromosome conformation capture techniques, such as Hi-C [54] and ChIA-PET [56], shed more lights on comprehensively understanding the changes in 3D genome organization. In the future, it will be interesting to determine the genome-wide changes in response to viral infection by integrating 3D genome methods with our current observations.

In addition, functional annotations of promoter-TSS regions combined with transcriptome analysis are critical to explore the distinct epigenetic signatures between BmN cells and BmNPV-infected cells. For instance, our results indicated that genes within the cell adhesion term lost accessibility and decreased expression during viral infection, suggesting that the host cells had a reduced ability to attach to the substrate. Indeed, our findings were supported by the cell detachment assay (**S2L Fig**). The pathogenicity of viruses normally causes cytotoxicity and tissue disruption, and viral envelope proteins have exerted essential functions in virulence and cell detachment [57, 58]. Therefore, it is necessary to investigate the roles of two major viral envelope proteins GP64 and Bm14 in virus-mediated cell detachment. Collectively, our data have fundamental implications for understanding the viral pathogenesis and baculovirus-insect interaction, which help to guide future baculovirus biology investigation and lepidoptera pest control.

To extend our insight into the changes of DNA accessibility in epigenetics, the relationships among the H3K4me3, H3K27ac, and H3K27me3 profiles were analyzed based on our ATAC-seq data. The public ChIP-seq data indicated that euchromatic markers (H3K4me3 and H3K27ac) were strongly correlated with the open chromatin regions, whereas heterochromatic modification (H3K27me3) showed less overlap, demonstrating that H3K4me3 and H3K27ac were necessary for maintaining chromatin accessibility in insect, *Bombyx mori*. Moreover, the levels of these modifications over the course of infection were examined by western blot analysis (**S5D Fig**). The results indicated that, H3K4me3 and H3K27me3 were markedly down-regulated at the very late stage of BmNPV infection, but H3K27ac was up-regulated, suggesting that the increase of H3K27ac as well as the decrease of H3K27me3 might contribute to the elevated DNA accessibility. Similar results have been reported for the Epstein-Barr virus (EBV) infected human primary B cells [59]. EBV infection induces a global decrease in facultative heterochromatic modifications and an increase in chromatin accessibility to endonucleases, indicating that these changes in host chromatin may be a universal mechanism of DNA viruses. In addition, our data not only demonstrated these changes, but also revealed the novel evidence that viral infection-induced increase in chromatin accessibility was closely associated with nucleosome disassembly and H3K27ac up-regulation. Therefore, further investigation is warranted to examine the functions of epigenetic modifications in BmNPV-infected cells, which is essential to reveal the molecular mechanisms of changes in chromatin organization.

To the best of our knowledge, this study has first reported the dynamic profiles of chromatin accessibility and architecture in response to baculovirus infection, which will help to provide a deeper insight into the virus-induced epigenomic regulation, and uncover this conserved mechanism from vertebrate to invertebrate DNA viruses.

## Materials and methods

### Cell culture, virus strain and infection

BmN cells were maintained as a monolayer in the Sf-900 II SFM medium (Invitrogen Life Technologies) supplemented with 3% fetal bovine serum (FBS, Invitrogen Life Technologies). The T3 strain of BmNPV was used as wild type (WT) virus in this study. Briefly, 5×10^6^ BmN cells were inoculated with WT-BmNPV (multiplicity of infection, MOI = 10) for 1 h at 27°C, then, the supernatant was replaced with fresh Sf-900 II SFM medium, and this time point was defined as 0 h p.i. Afterwards, cells were harvested at indicated time points (8, 24, and 48 h p.i.) for subsequent ATAC-seq, RNA-seq and biochemical assays.

### Immunofluorescence (IF)

Briefly, cells on the coverslips were infected with WT virus at an MOI of 10. Thereafter, cells were fixed with 4% paraformaldehyde for 15 min, permeabilized with 0.1% TritonX-100 for 20 min, blocked in the PBS solution containing 5% BSA for 1 h, and incubated with primary rabbit polyclonal anti-Histone H3 antibody (Abcam) and mouse monoclonal anti-Lamin Dm0 antibody (DHSB) overnight at 4°C at indicated time points. Afterwards, cells were incubated with secondary Alexa 488-conjugated goat anti-rabbit antibody and Alexa 546-conjugated goat anti-mouse antibody (Invitrogen). Finally, cells were incubated with 4’,6-diamidino-2-phenylindole (DAPI, Beyotime), and examined through ZEISS LSM 780 confocal scanning laser microscopy (CSLM).

### Transmission electron microscopy (TEM)

High-pressure frozen (HPF) and freeze substitution (FS) was performed in combination with TEM, as previously reported [60]. In brief, uninfected BmN cells and BmNPV-infected cell pellets at 24 and 48 h p.i. were placed into the copper carriers filled with hexadecane, and then the samples were vitrified using a high-pressure freezer EMPACT_2_ (Leica). Afterwards, the vitrified cells were preserved in liquid nitrogen and later placed in the FS cocktail flasks at -90°C in the Automate Freeze-Substitution (AFS). The substitution medium was comprised of 2% OsO_4_ and 2.5% H_2_O in acetone, and FS was carried out in accordance with the following procedure: -90°C for 24 h; heating to -60°C at a rate of 2°C/h; -60°C for 8 h; heating to -30°C at a rate of 2°C/h; -30°C for 8 h; and heating in pure acetone for thrice at an interval of 10 min. Finally, the samples were progressively embedded within the epoxy-acetone mixture (1:3) for 2 h at -30°C before heating to 0°C at a rate of 15°C/h; then within the epoxy-acetone mixture (1:1) for 2 h at room temperature; and eventually within the epoxy/acetone mixture (3:1) overnight at room temperature. Later, samples were incubated in pure epoxy for 2 h thrice, and finally in a new bath of pure epoxy overnight prior to polymerization at 60°C for 24 h. Thereafter, the 70-nm ultrathin sections were cut using a diamond knife (45° angle) on the Leica UC6 ultramicrotome, and collected onto the carbon-coated grids. Eventually, the samples were observed at room temperature using the TEM Tecnai G2 spirit at 120 kV.

### Preparation and sequencing of ATAC-seq, ChIP-seq and RNA-seq libraries

ATAC-seq libraries were prepared as described previously [17]. In brief, 5×10^4^ cells were lysed in 50 μl cold lysis buffer (containing 10 mM Tris-HCl, pH 7.4, 10 mM NaCl, 3 mM MgCl2 and 0.1% IGEPAL CA-630) to obtain the nuclei for each sample. Later, the Nextera DNA Sample Preparation Kit (Illumina) was adopted for carrying out the Tn-5 transposition reaction at 37°C for 30 min. Then, DNA fragments were purified through the MinElute Kit (Qiagen), and amplified for 5 cycles using those previously designed primers containing both compatible adaptors and barcodes. Subsequently, the resulting ATAC-seq libraries were purified (MinElute Kit, Qiagen), and the final libraries were sequenced on the NovaSeq 6000 illumina platform with a paired-end read of 150 bp.

ChIP-seq libraries were prepared as described previously [41]. Cells were collected and cross-linked with 1% formaldehyde for 10 min at 25°C and then quenched by addition of glycine to a final concentration of 125 mM. Afterwards, samples were lysed and chromatin was obtained on ice. Chromatin was sonicated to get soluble sheared chromatin (average DNA length of 200–500 bp). Human HeLa cell chromatin was prepared by same method. Then mixed HeLa and BmN cell chromatin in ration of one to four. The samples were immunoprecipitated by anti-H3K27ac (Abcam) antibody. Later, the libraries were prepared using NEXTflex ChIP-Seq Library Prep Kit (Bioo Scientific) for Illumina Sequencing according to the manufacturer’s instructions. The final libraries were sequenced on the HiSeq X ten illumina platform with a pair-end read of 150 bp.

Afterwards, the total RNA was isolated from the harvested cells using RNAiso Plus (Takara) according to the manufacturer instructions. Three microgram of RNA per sample was used as the input material for library preparation, the RNA-seq libraries were constructed using the NEBNext Ultra RNA Library Prep Kit for Illumina (NEB), and index codes were added to attribute the sequences to each sample. Then, the as-obtained RNA-seq libraries were sequenced on the HiSeq X ten illumina platform with a paired-end read of 150 bp.

### Primary processing of ATAC-seq, ChIPseq and RNA-seq data

ATAC-seq data were processed using the Kundaje Lab’s ATAC-seq pipeline [61] (including quality control, trimming, filtering, aligning and peaks calling) after mild modifications. In brief, the raw sequencing reads were initially processed by the FastQC program for quality control, and later the sequencing adaptors and poor-quality reads were removed using Trimmomatic [62]. Afterwards, the filtered reads were mapped to the reference *Bombyx mori* genome and BmNPV genome using BWA [63], respectively. Then, the sam files were converted into bam format using Samtools [64]. MACS2 [65] was adopted for peaks calling, and an initial threshold q-value of 0.01 was used as the cutoff value. Then, the ATAC-seq data were normalized using the stably expressed genes from RNA-seq. Firstly, 30 stably expressed genes were screened from all samples with low variance; secondly, the fragments in region within 1kb of the transcript start sites of those 30 genes were counted from all ATAC-seq samples, and then the ATAC-seq data in bam format were normalized, so that these fragment counts were basically the same among all samples. The read count data were visualized by converting the raw bam files to the bigwig files using the IGV tool [66].

The raw ChIP-seq reads were trimmed by Trimmomatic [62] and then the filtered reads were aligned to the reference *Bombyx mori* genome and human genome using BWA [63]. The sam files were converted into bam format using Samtools [64]. For data scaling, ChIPseqSpikeInFree [67] was used to normalize the ChIP-seq according to the spike-in control. MACS2 [65] was adopted for peaks calling using the default parameters. In addition, the public ChIP-seq data generated from BmN cells were analyzed by the same method except for spike process.

Subsequently, the raw RNA-seq read quality was evaluated by the FastQC program, and the sequencing adaptors were trimmed by Trimmomatic [62]. Later, the filtered reads were aligned to the reference *Bombyx mori* genome and BmNPV genome using STAR [68], and only the uniquely mapped reads were preserved, while the BmNPV reads were removed. The read counts and RPKM were calculated by HTSeq [69].

### Analysis of differential accessible peaks and K-means clustering

To generate a consensus set of unique peaks, two replicate ATAC-seq peaks were merged according to the distance between proximal end of < 1bp. Then, the average score of bigwig was further calculated across each peak using the UCSC tool [70]. Finally, the R package Limma [26] was employed to identify those differential accessible peaks between two different groups, with the threshold fold change (FC) of > 2 and p-value of < 0.05.

Moreover, the ATAC-seq peaks of all samples were also merged according to the distance between proximal end of <1bp. Later, the UCSC tool was adopted to calculate the average score of bigwig across all peaks [70], whereas the K-means algorithm was utilized to cluster the peaks. Heatmap revealed that the peaks were clustered in all samples using deeptools2 [71].

### Genome features and Gene Ontology (GO) analysis

HOMER [25] was employed to associate ATAC-seq peaks with the nearest genes. Typically, ATAC-seq peaks were assigned into five categories, including promoter-transcription start site (TSS), transcription terminal site (TTS), exon, intron and intergenic. Of them, promoter was defined as the region within 3kb of the reference TSS, as determined by the UCSC tool.

For functional analysis, GO annotations were downloaded from agriGO (http://bioinfo.cau.edu.cn/agriGO/download.php). The significant GO categories were identified through Fisher’s exact test, and FDR was adopted to correct the p-values.

### Principal component analysis (PCA)

PCA plots were generated based on the normalized read counts against all merge peaks. Besides, these read counts were normalized to the sequence depth before they were adopted for PCA using the PRCOMP function of R language.

### Cell detachment assay

For the cell detachment assay, cells were infected with WT virus at an MOI of 10. Then, the mediums containing floating cells were collected at indicated time points, and later those attached cells were trypsinized and harvested. At the same time, the collected cell solutions were dyed with trypan blue, and only those undyed cells were counted with the cell counting board. Next, the percentage of detached cells was calculated according to the formula below: detached cells (%) = (floating cells/ (floating cells + adherent cells) × 100%).

### Immunoblotting

For immunoblotting assay, 30 μg cellular proteins at indicated time points were mixed with 2× Laemmli buffer, separated through 12% sodium dodecyl sulfate polyacrylamide gel electrophoresis (SDS-PAGE), and examined through western blotting using the rabbit polyclonal anti-Histone H3 antibody (Abcam), rabbit polyclonal anti-α-tubulin antibody (Beyotime), mouse monoclonal anti-GP64 antibody (Sigma), rabbit polyclonal anti-H3K4me3 antibody (Abcam), rabbit polyclonal anti-H3K27ac antibody (Abcam), and rabbit polyclonal anti-H3K27me3 antibody (Active motif).

### Quantitative real-time PCR (qRT-PCR) analysis

For qRT-PCR analysis, BmN cells and BmNPV-infected cells at indicated time were harvested and total RNA was extracted by using TRIzol reagent (Invitrogen). 5μg total RNA was used to synthesis the first-strand cDNAs by TransScript One-Step gDNA Removal and cDNA Synthesis SuperMix (TransGen). qRT-PCR was performed using Hieff qPCR SYBR Green Master Mix (Yeasen) and the primers of qRT-PCR were used as follows: *Tua2*-F: 5’-TGCCCGAGGACACTATACCA-3’, *Tua2*-R: 5’- ACACGAGGAAACCCTGAAGC-3’, *asf1*-F: 5’-CGCCAATGGAATGCTGAGATTAT-3’, *asf1*-R: 5’-GTACAAACAAAGCCTTGAAAACGG-3’, *cbx5*-F: 5’-ATCCATTGGAAGGGCTGGTC-3’, *cbx5*-R: 5’-GGCGCTGCTCACTTTATCCA-3’.

### In vivo Terminal-deoxynucleoitidyl Transferase Mediated Nick End Labeling (TUNEL) and DNase I & TUNEL assays

In vivo DNase I & TUNEL assays were carried out as described previously [36] with mild modifications. In brief, cells on the coverslips were infected with WT virus at an MOI of 1, and fixed with 4% paraformaldehyde, permeabilized in 0.1% TritonX-100, blocked with the PBS solution containing 5% BSA, and incubated with primary rabbit polyclonal anti-VP39 antibody at indicated time points. Afterwards, cells were subsequently incubated with secondary Alexa 546-conjugated goat anti-rabbit antibody (Invitrogen), and then treated with DNase I for 5 min at 27°C. Then, TUNEL assays were performed using the One Step TUNEL Apoptosis Assay Kit (Beyotime) in accordance with manufacturer protocols. Finally, cells were incubated with DAPI (Beyotime), and examined with the ZEISS LSM 780 confocal scanning laser microscopy (CSLM). The procedures for TUNEL assay were same as those mentioned above, except for the DNase I treatment.

### Statistical analysis

Pearson’s correlation analysis was performed with the R platform. Student’s t-test and two-way ANOVA were conducted using the GraphPad Prism6. P<0.05 indicated statistical significance.

### Accession numbers

The ATAC-seq, RNA-seq and ChIP-seq data generated in this study have been submitted to the NCBI BioProject database (https://www.ncbi.nlm.nih.gov/bioproject/) under accession number PRJNA549316 and PRJNA628845. The histone modifications ChIP-seq data were obtained from the NCBI Sequence Read Archive (SRA) database (https://www.ncbi.nlm.nih.gov/sra) under accession number DRX001335, DRX016832 and DRX016833.

## Supporting information

S1 Fig ATAC-seq data quality metrics and reproducibility. **(A)** The fragment length histograms of the two replicates of BmN cells indicate the expected pattern of nucleosomal transposase insert sizes. **(B)** Scatter plots showing the ATAC-seq reads correlation coefficient between replicates of each group. **(C)** IGV genome browser view of *eIF 2a kinase* gene ATAC-seq signal profiles.
(TIF)

S2 Fig Differentially accessible peak analysis. **(A)** Distribution of genomic features of opened or closed peaks during BmNPV infection. **(B)** Volcano plot of differentially accessible peaks between BmN cells with 8 and 24 h p.i. group. **(C)** Cumulative distribution function of peak accessibility changes between BmN cells and the 8 h p.i. group. **(D)** Cumulative distribution function of peak accessibility changes between BmN cells and 24 h p.i. group. **(E)** Cumulative distribution function of peak accessibility changes between BmN cells and 48 h p.i. group. **(F)** Normalized ATAC-seq profiles at *LOC101735468* loci. Shaded regions are representative of an increase at 8 h p.i.
(TIF)

S3 Fig Gene ontology analysis of differentially accessible peaks. **(A-F)** Biological processes (GO terms) enriched in genes which correspond to gained at 8 h p.i., lost at 8 h p.i., gained at 24 h p.i., lost at 24 h p.i., gained at 48 h p.i., and lost at 48 h p.i., respectively.
(TIF)

S4 Fig K-means cluster analysis of all peaks. **(A-C)** Biological processes (GO terms) enriched in genes which correspond to cluster I, cluster II and cluster IV, respectively. **(D, E)** Normalized ATAC-seq profiles at *RpL21*and *E2f1* loci, respectively. Shaded regions represent increase during BmNPV infection. **(F, G)** Boxplots of G-protein coupled receptor signaling pathway and proteolysis associated gene expression in all groups. Two-way ANOVA was performed between BmN and 48 h p.i. groups. **(H)** The heatmap shows the expression of genes related to nucleosome assembly GO term during BmNPV infection.
(TIF)

S5 Fig The relationships between histone modifications and chromatin accessibility in BmN cells. **(A-C)** Venn diagrams showing the overlaps between histone modifications and ATAC-seq peaks in BmN cells. **(D)** BmN cells and BmNPV-infected cells were analyzed by western blot with the indicated histone modifications antibodies.
(TIF)
