# Supplementary figures and images for: Dynamic chromatin accessibility profiling reveals changes in host genome organization in response to baculovirus infection
Source: PLoS Pathog. 2020 Jun 8;16(6):e1008633. doi: 10.1371/journal.ppat.1008633 (PMC7326278; doi:10.1371/journal.ppat.1008633)

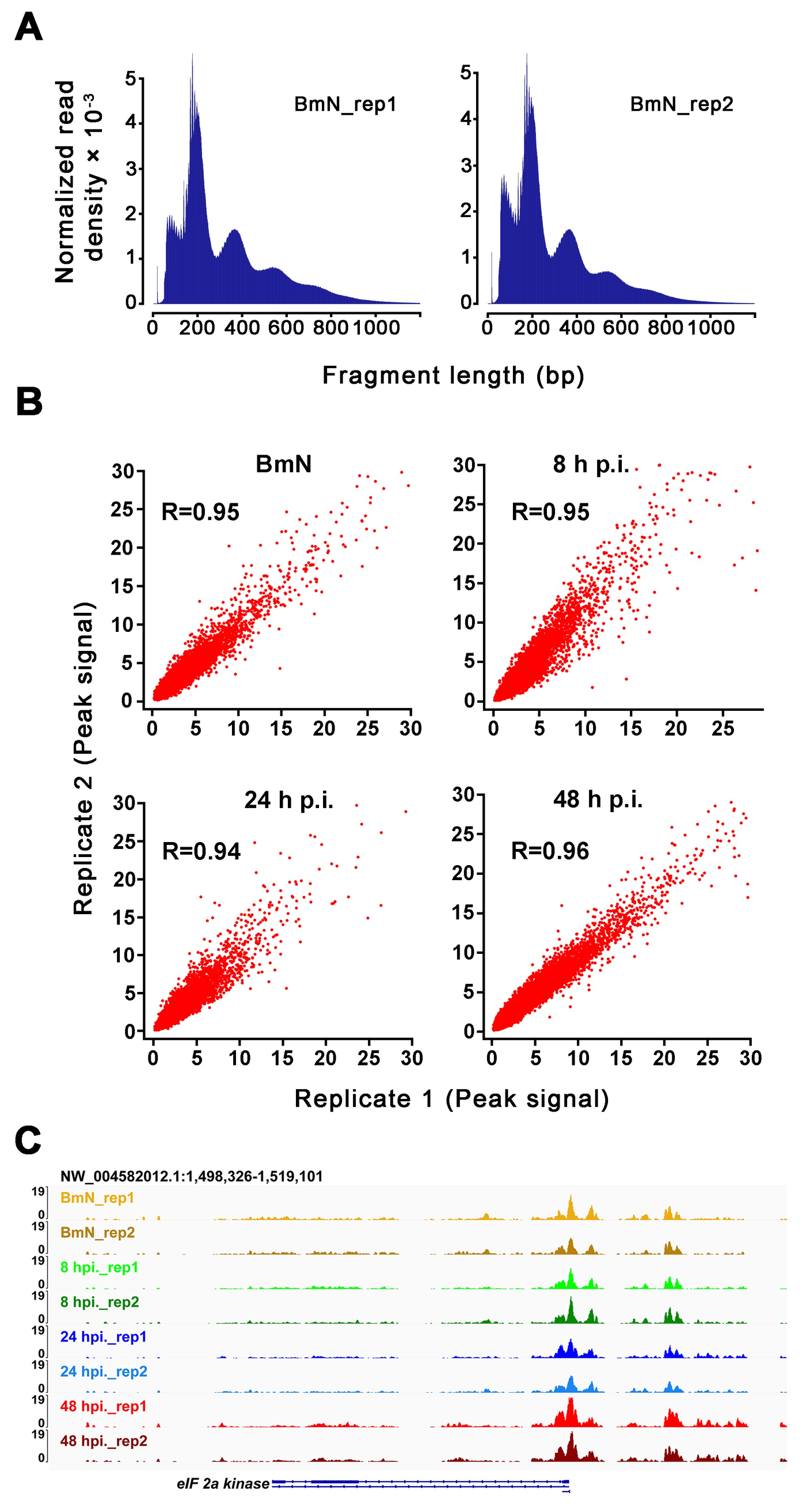

Supplement: S1 Fig — (A) The fragment length histograms of the two replicates of BmN cells indicate the expected pattern of nucleosomal transposase insert sizes. (B) Scatter plots showing the ATAC-seq reads correlation coefficient between replicates of each group. (C) IGV genome browser view of eIF 2a kinase gene ATAC-seq signal profiles. (TIF) [file ppat.1008633.s001.tif]

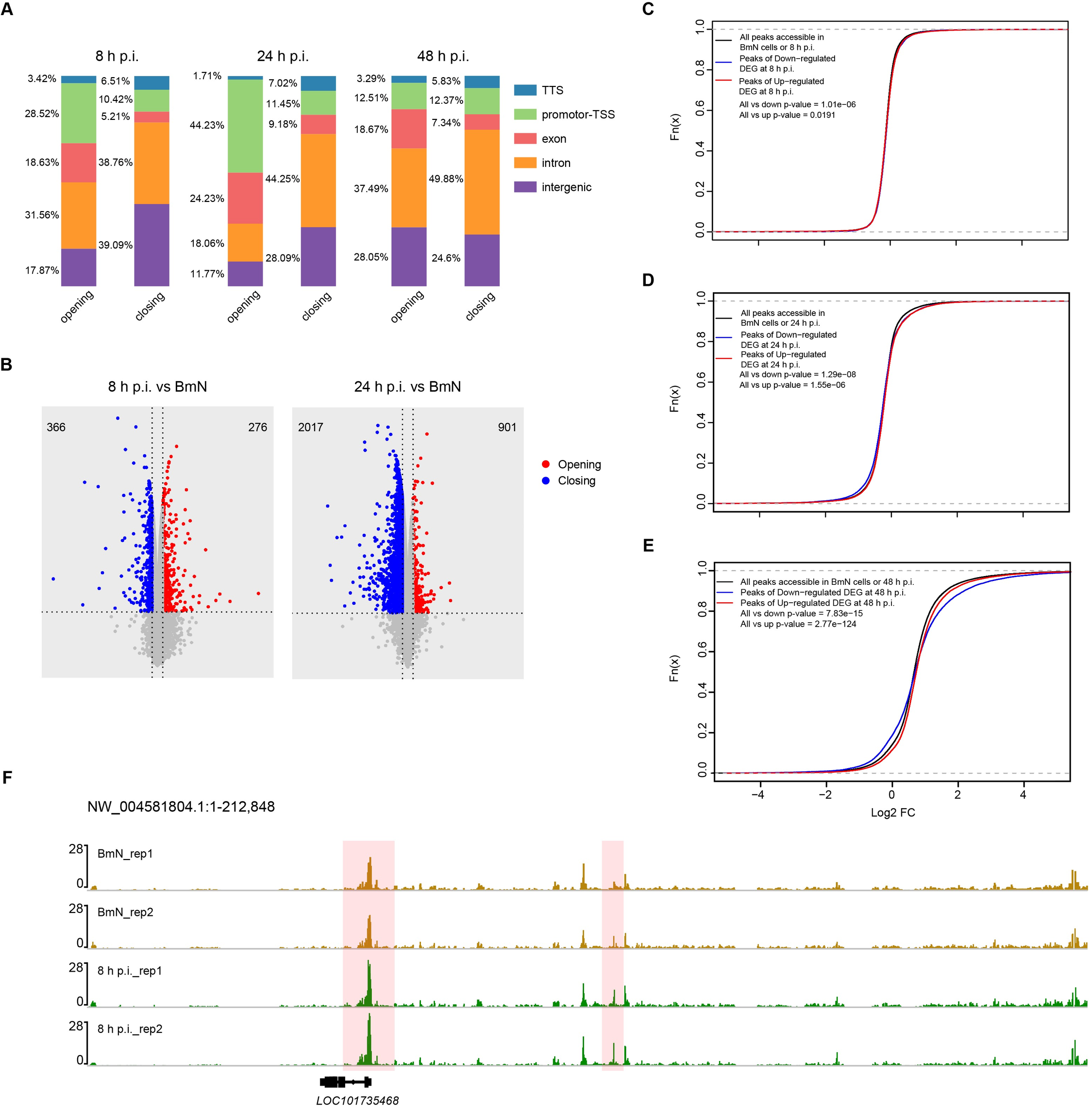

Supplement: S2 Fig — (A) Distribution of genomic features of opened or closed peaks during BmNPV infection. (B) Volcano plot of differentially accessible peaks between BmN cells with 8 and 24 h p.i. group. (C) Cumulative distribution function of peak accessibility changes between BmN cells and the 8 h p.i. group. (D) Cumulative distribution function of peak accessibility changes between BmN cells and 24 h p.i. group. (E) Cumulative distribution function of peak accessibility changes between BmN cells and 48 h p.i. group. (F) Normalized ATAC-seq profiles at LOC101735468 loci. Shaded regions are representative of an increase at 8 h p.i. (TIF) [file ppat.1008633.s002.tif]

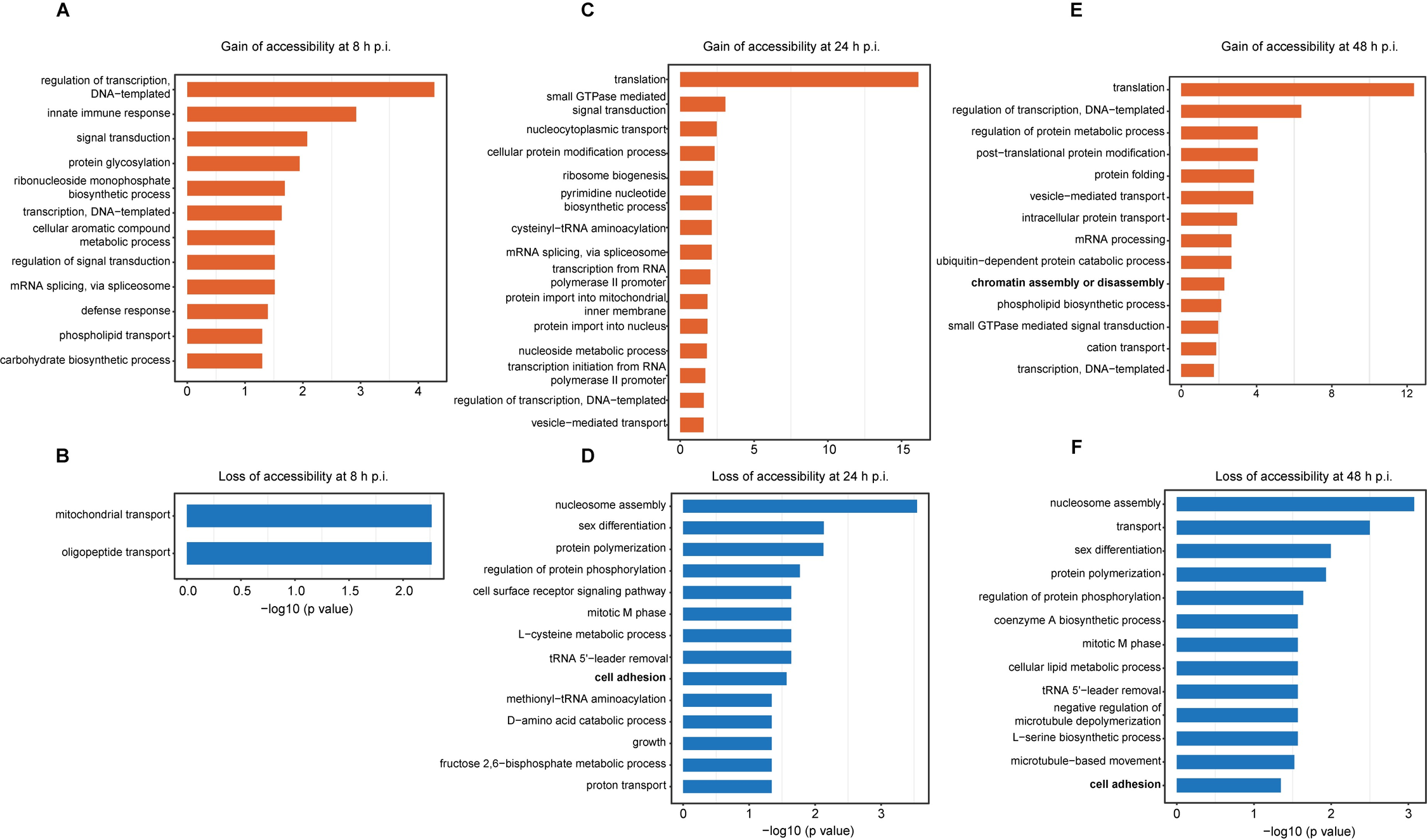

Supplement: S3 Fig — (A-F) Biological processes (GO terms) enriched in genes which correspond to gained at 8 h p.i., lost at 8 h p.i., gained at 24 h p.i., lost at 24 h p.i., gained at 48 h p.i., and lost at 48 h p.i., respectively. (TIF) [file ppat.1008633.s003.tif]

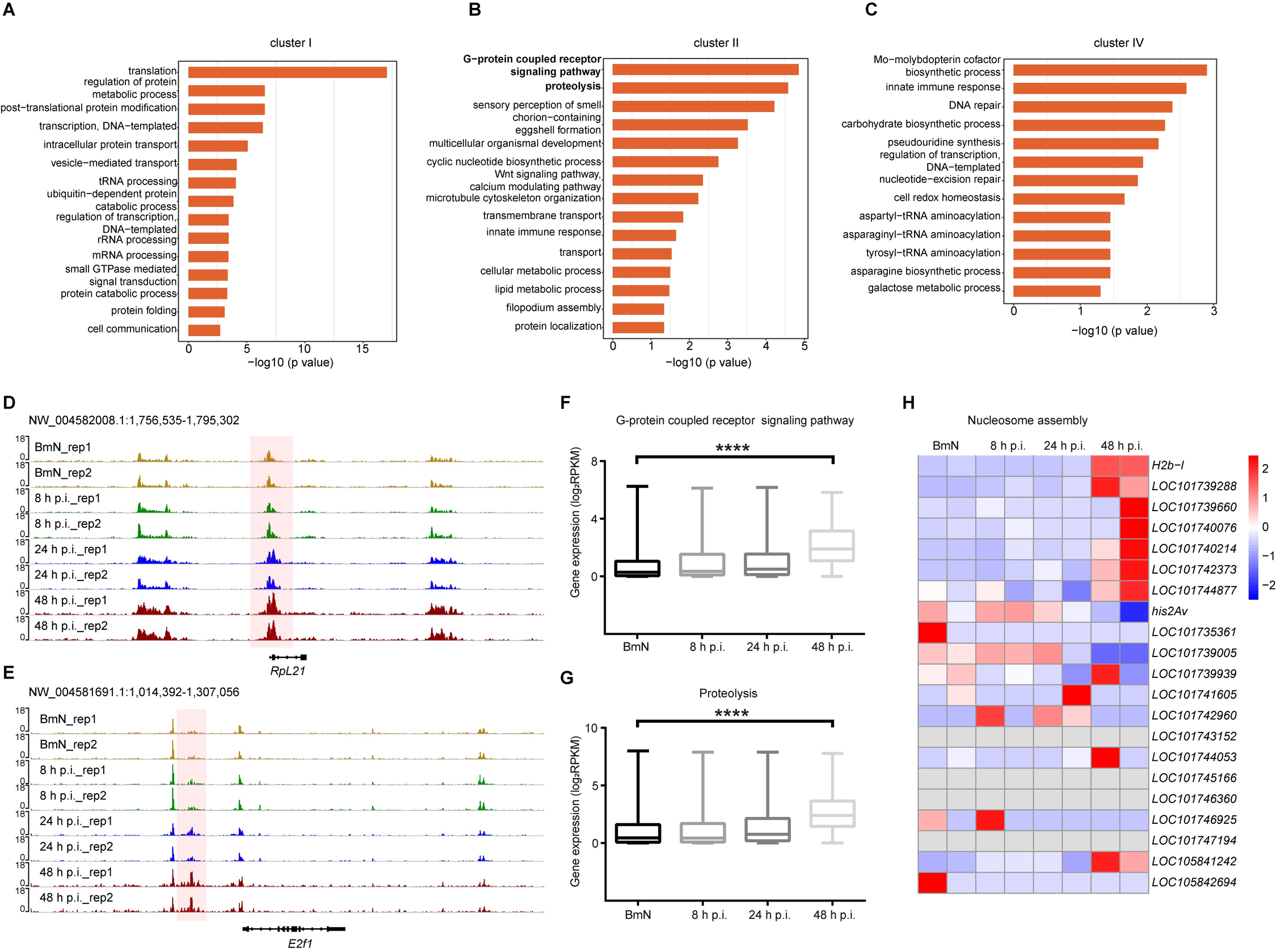

Supplement: S4 Fig — (A-C) Biological processes (GO terms) enriched in genes which correspond to cluster I, cluster II and cluster IV, respectively. (D, E) Normalized ATAC-seq profiles at RpL21and E2f1 loci, respectively. Shaded regions represent increase during BmNPV infection. (F, G) Boxplots of G-protein coupled receptor signaling pathway and proteolysis associated gene expression in all groups. Two-way ANOVA was performed between BmN and 48 h p.i. groups. (H) The heatmap shows the expression of genes related to nucleosome assembly GO term during BmNPV infection. (TIF) [file ppat.1008633.s004.tif]

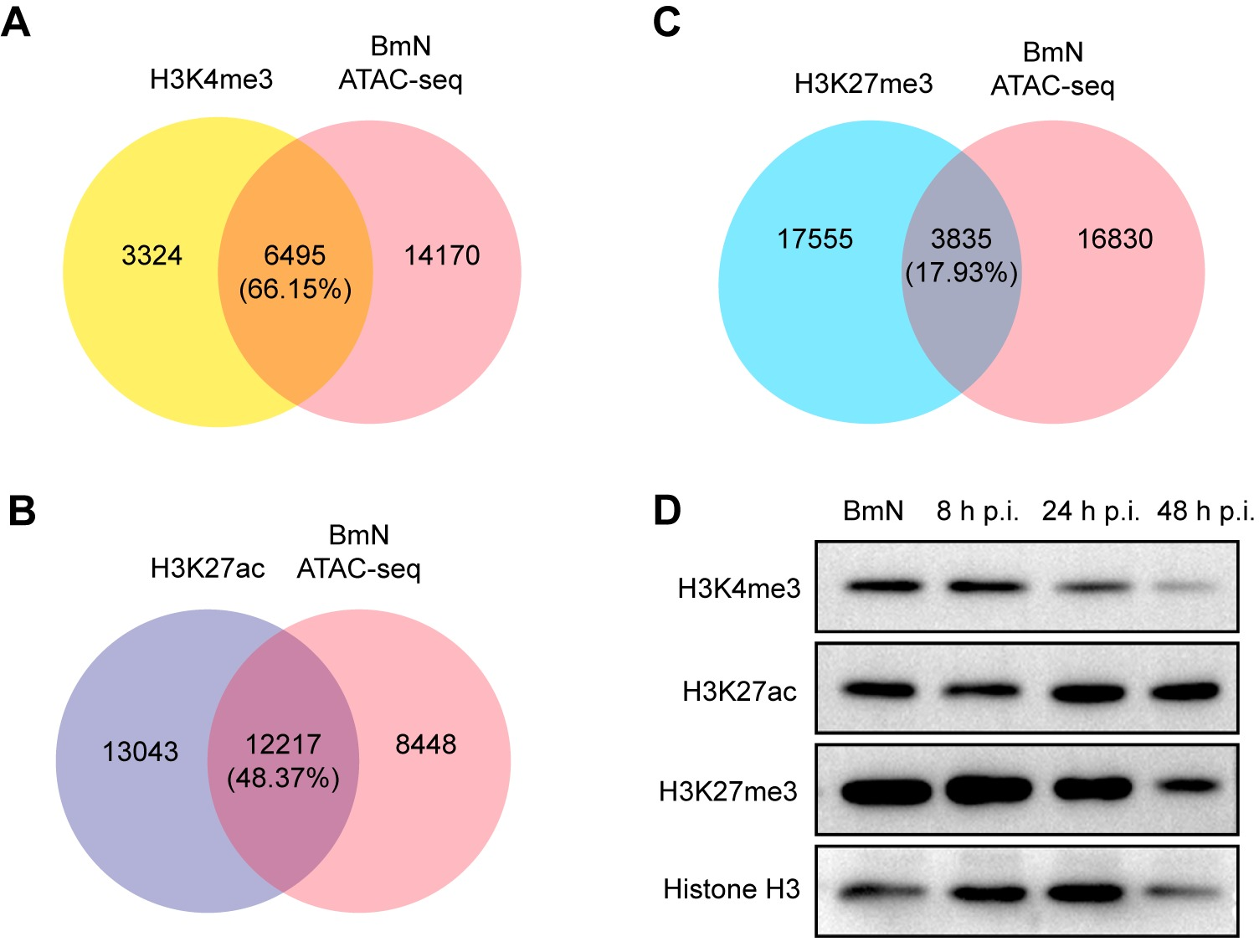

Supplement: S5 Fig — (A-C) Venn diagrams showing the overlaps between histone modifications and ATAC-seq peaks in BmN cells. (D) BmN cells and BmNPV-infected cells were analyzed by western blot with the indicated histone modifications antibodies. (TIF) [file ppat.1008633.s005.tif]
